# Milk Instead of Medicine

**Published:** 1893-07

**Authors:** 


### MILK INSTEAD OF MEDICINE.

Wise physicians always prescribe a diet instead of a drug for a patient whenever his illness can be cured by food alone. The food is one of the most important factors in moulding the life of an individual; both the mind and body require it for their best development. We too seldom realize that much of our bodily discomforts arise from having had an insufficiency of nourishing food. We stoutly deny being underfed when our doctor says, "You need more food." Six meals a day would barely supply fuel enough to keep the fire burning in the average American woman or man of to-day! The breadwinner of the family comes home from his business at night too tired, too nervous to eat. Very possibly he has not tasted food all day since he ate a hasty breakfast of a roll and a cup of coffee.

Is it any wonder such a man is irritable and soon becomes a sufferer from nervous prostration? The lack of and insufficiency of nutritious food puts a human being in a condition to die of any complaint. It is not the well-fed that die of consumption—it is they that have no time for eating or resting. The healthiest and longest lived are those that have leisure enough to eat their meals and do eat them. Food keeps the blood vessels full of good blood—disease germs floating about cannot find a lodging place in well nourished persons.

Tempting choice viands are not within the reach of every purse—good, simple, wholesome food is. The poorest man can afford to drink milk, and milk contains every essential needful for the sustenance of vitality and the restoration of lost powers. There are so many ways of preparing milk either alone or in combination with eggs, fresh vegetables, as in soups, etc., that one cannot exclaim at the monotony.

First of all try boiled milk, bearing in mind that milk may be contaminated and that boiling effectually ends the possibility of danger from it. If cold milk is more grateful than hot, drink it cold, taking care to have no ice in direct contact with it. Put the milk in bottles or kettles and let these be in contact with the ice. Cultivate the habit of drinking eight or ten glasses of milk every day. If this is done it will be safe enough to omit meals occasionally. Milk does not seem to agree with some few persons, and for them three or four ounces daily of cream will prove a most excellent food. Hot milk is more effective in relieving nervousness and fatigue than any alcoholic preparation, and is far less expensive. Many "incurable" maladies may be put to flight by living on a milk diet—in ten days one will be improved, and in a few months will find health fully restored.

